# Spontaneous Tumor Lysis Syndrome in Metastatic Melanoma

**DOI:** 10.4021/wjon347w

**Published:** 2011-08-24

**Authors:** Mingchen Song, Chris C. W. Chan, David A. Stoeckel

**Affiliations:** a Division of Pulmonary, Critical Care and Sleep Medicine, Saint Louis University, St. Louis, MO 63104, USA

**Keywords:** Spontaneous tumor lysis syndrome, Metastatic melanoma

## Abstract

Tumor lysis syndrome (TLS) complicating non-hematologic malignancy is infrequent and spontaneous TLS is a very rare occurrence in patients with solid tumors. We report a case of spontaneous TLS in a patient with metastatic melanoma. Clinicians should have awareness of the possibility of spontaneous TLS in patients with solid tumors and should recognize the clinical presentation and laboratory tests for its diagnosis.

## Introduction

Tumor lysis syndrome (TLS) is a well-known oncologic emergency that is characterized by severe metabolic derangements. As the consequence of tumor dissolution, TLS causes the massive release of intracellular components into the blood. Typically, TLS occurs in patients with hematologic malignancies such as non-Hodgkin lymphoma and leukemia who are exposed to chemotherapy, radiation, or corticosteroids. However, spontaneous TLS in the absence of treatment has been described, albeit rarely in patients with solid tumors. In this context, sporadic cases of TLS in metastatic melanoma have reported in the literature. To our knowledge, acute spontaneous TLS has not been previously reported with metastatic melanoma.

## Case Report

A 46-year-old man without significant past medical history was found to have a skin lesion in the left lower abdominal wall. Excision was performed and pathology disclosed malignant melanoma (nodular type, Clark’s level V; Breslow’s thickness 5.5 mm). A PET/CT scan one month later showed multiple FDG-avid soft tissue lesions in the chest, abdominal subcutaneous skin, and left groin areas. Subsequently, the patient underwent wide excision of abdominal wall and lymph node dissection. Pathology revealed malignant melanoma with groin and internal mammary lymph node involvement.

Three months later, the patient was admitted to the hospital with progressive abdominal pain associated with nausea and vomiting for three days duration. Vital signs on presentation included a temperature of 36.1°C, pulse of 116 beats/min, respiratory rate of 24/min, and blood pressure of 99/77 mmHg. Physical examination was remarkable only for the abdominal wall surgical scar, dehydration, and generalized weakness. The circulating white blood cells were 14,800 cells/mL, hemoglobin was 18.46 g/dL, and the platelet count was 155,000 cells/mL. Chemistry panel showed a potassium level of 5.0 mmol/L, hypochloridemia (chloride, 90 mmol/L), elevated alkaline phosphatase of 628 U/L, alanine aminotransferase, 165 U/L, and aspartate aminotransferase, 922 U/L. Serum bicarbonate was mildly reduced (20 mmol/L), BUN was 61 mg/dL and creatinine, 1.1 mg/dL. Serum lipase was normal at 57 units/L as was total calcium (8.6 mg/dL). CT scan of the chest, abdomen and pelvis with contrast demonstrated widespread metastasis to the liver and spleen.

**Figure 1:**
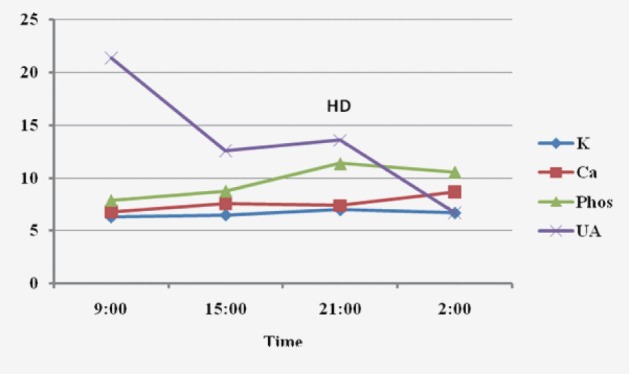
Biochemical parameters within the last 24 hours prior to death. K: potassium (mmol/dL); Ca: calcium (mg/dL); phos: phosphorrus (mg/dL); UA: uric acid (mg/dL); HD: hemodialysis.

Upon admission, the patient was aggressively hydrated intravenously. His condition, however, progressively deteriorated. On day 3 after admission, hyperkalemia (6.3 mmol/L), hyperphosphatemia (7.9 mg/dL), hypocalcaemia (6.8 mg/dL), and hyperuricemia (21.4 mg/dL) were noted in conjunction with worsening creatinine (1.6 mg/dL) and markedly elevated LDH (5,200 U/L). He was transferred to the intensive care unit because of progressive dyspnea, intubated, and placed on mechanical ventilation. Vasopressor support with norepinephrine was initiated because of arterial hypotension and oliguria. A diagnosis of spontaneous TLS was made based on the biochemical profile and subsequently hemodialysis was started. Although the serum uric acid concentration decreased with hemodialysis, hyperkalemia and hyperphosphatemia persisted (Fig. 1). Despite full therapeutic measures he expired due to cardiac arrest within 24 hours.

## Discussion

Tumor lysis syndrome is a catastrophic condition that occurs due to the rapid destruction of tumor cells with massive release of cellular breakdown products, typically seen following the treatment of malignancies [1]. The syndrome is characterized by the rapid development of hyperuricemia, hyperkalemia, hyperphosphatemia, hypocalcaemia, metabolic acidosis and acute renal failure [1]. The electrolyte imbalances and severe metabolic acidosis can lead to serious and potentially fatal arrhythmias [1].

Most commonly, TLS is associated with hematologic malignancies such as non-Hodgkin lymphoma and leukemia. However, a variety of solid tumors have been rarely associated with the syndrome, including breast carcinoma, neuroblastoma, small cell lung carcinoma, germ cell tumor, thymoma, soft tissue sarcomas, ovarian carcinoma, head and neck squamous carcinomas. TLS usually follows treatment with chemotherapy, radiation and/or corticosteroids, but it can even occur spontaneously in the absence of treatments.

Traditionally, malignancies such as metastatic melanoma are relatively resistant to chemotherapy and prophylaxis of TLS is not routine in clinical practice. However, new biochemotherapeutic regimens for metastatic melanoma have been shown to achieve more effective tumor regression. TLS in such treated melanoma has been reported in 7 patients (Table 1) [2-8]. All had bulky metastatic disease with large tumor burdens. One case occurred after cisplatin chemotherapy and embolization [7]; another after biotherapy [2]. Three cases developed after combined biotherapy and chemotherapy [3, 4, 6]; and 2 cases after corticosteroid treatment [5, 8]. In striking contrast, our case developed spontaneously. To our knowledge, this is the first report of spontaneous TLS occurring in non-treated metastatic melanoma. TLS in metastatic melanoma carries a dismal prognosis; the outcome usually is fatal and all cases including ours died, with one exception.

**Table 1:** Characteristics of Tumor Lysis Syndrome (TLS) in Metastatic Melanoma

Reference	Age, sex	Primary site	Metastatic lesions	Trigger	Onset of TLS	Hemodialysis	Outcomes
2	76, M	NS	Liver, lung, spleen, pelvis, lymph nodes	IFN-α, anti-GD3 ganglioside monoclonal antibody	8 h	Not required	Died
3	61, M	Abdomen	Liver, lung, axillary lymph node	IL-2, IFN-α, cisplatin, dacarbazine, vinblastine	24 h	Not required	Died
4	41, M	NS	Liver, GI tract	TNF-α, cisplatin, dacarbazine	2 days	Initiated, but discontinued at patient’s own request in a week	NS
5	56, F	Shoulder	Liver, lung, bone	Hydrocortisone	7 h	Not required	Died
6	36, F	Back	Liver, lung	IL-2, IFN-α, cisplatin, dacarbazine, vinblastine	3 days	Initiated with improvement of renal dysfunction	Died
7	62, M	Choroid	Liver	Transcatheter arterial infusion of cisplatin and embolization	24 h	Initiated with improvement of renal dysfunction	Died
8	42, M	NS	Liver, spleen, bone, Abdominal lymph nodes	Corticosteroid	2 days	NS	Died
This case	46, M	Abdomen wall	Liver, spleen, lymph node	No	Spontaneous	Required	Died

TNF- α, tumor necrosis factor; IL-2, interleukin-2; IFN-α, interferon-α; NS, not stated.

Overall, the risk factors for TLS include a high tumor proliferation rate, large tumor burden, chemo-sensitivity and increased circulating LDH concentrations. Interestingly, all 7 cases including ours had liver metastasis. We currently do not know whether this latter fact simply indicates a large tumor burden or constitutes another risk factor. Moreover, the exact mechanism by which the catastrophic state developed in our patient is unclear, although he had most risk factors – large tumor burden, high tumor proliferation rate and elevated serum LDH concentration. Conceivably, prerenal azotemia caused by hypovolemia and renal vasoconstriction precipitated acute renal failure contributed to the occurrence of spontaneous TLS in our patient.

In summary, acute spontaneous TLS should be considered in the differential diagnosis of critically ill patients with malignancy and acute renal dysfunction in the setting of characteristic biochemical abnormalities. Equally critical is the recognition of patients at risk and the effective use of prophylactic therapy to prevent this catastrophic syndrome.
